# Assessing multiple free-roaming dog control strategies in a flexible agent-based model

**DOI:** 10.1038/s41598-023-47076-x

**Published:** 2023-11-14

**Authors:** A. J. Yoak, K. M. Calinger, E. Hiby

**Affiliations:** 1 Otterbein University, Westerville, OH USA; 2 The Ohio State University, Columbus, OH USA; 3 International Companion Animal Management Coalition, Cambridge, UK

**Keywords:** Ecological modelling, Population dynamics, Computational models

## Abstract

Management of free-roaming dog populations is required to mitigate the threat of pathogens like rabies, minimize conflict with people, wildlife, and livestock and improve dog welfare however there are multiple strategies currently employed including sterilization, vaccination, and lethal removal. This work describes an agent-based stochastic model, ‘StreetDogSim’ that can be used as a planning tool to investigate the predicted impact of different strategies with variable implementation approaches and adjustable parameters to match local conditions. Here, we explore the effects of different management strategies with additional variation in their duration, intensity, and vaccine quality on important population metrics such as overall size, demographics, vaccination coverage, time until effective population suppression, and duration of suppression. Under most model parameterizations, a strategy that targets females for sterilization with vaccination outperforms all other options with respect to population control and demographic changes.

## Introduction

Domestic dogs have evolved alongside humans for thousands of years and are ubiquitous as companion and working animals globally. Current global estimates of dog population size range from 687 million^1^ to 987 million^2^ with especially high densities in urban environments^3^.

Although most dogs are valued by their caretakers, dogs can also cause problems, especially when free-roaming^4,5^. Negative impacts include disease transmission, dog bites, road traffic accidents, predation of livestock and wildlife, noise, and fecal pollution of the environment^6–11^. Canine welfare is also a significant concern, including high puppy mortality, with estimates of survival as low as 18–25% for dogs below the age of 1 year in India^12,13^. Management of dog populations to mitigate these risks to communities, and the dogs, is therefore a global challenge and is the subject of international standards^14^ and guidelines^15^. What measures to use within dog population management (DPM) interventions depend on local dog population dynamics as well as human knowledge, attitudes, and practices.

In India, the Animal Birth Control (Dog) Rules^16^ stipulate that rabies control and DPM should be achieved by catching dogs then sterilizing and vaccinating them before returning them to the point of capture; this is also termed Catch, Neuter, Vaccinate and Return or CNVR^14^. This technique is appropriate for free-roaming dog populations that are well tolerated and fed regularly by local communities. Such dogs may be termed ‘community dogs’ and will have relatively high breeding success and the potential for good welfare whilst living on the streets. This would not be an appropriate approach in communities where roaming dogs are not well tolerated^15^.

Populations of community dogs are common in South and South East Asia and success of CNVR has been reported in multiple locations including but not limited to: reductions in rabies^20–22^, reductions in dog bites^23^, reductions in dog population size^24^, improved welfare^25,26^ and economic benefits^27^. However, CNVR has also produced mixed results when applied to cats^28–32^ and has been insufficient for managing dog populations where abandonment of owned dogs is the predominate source of free-roaming dogs^18,19^. CNVR alone is unable to influence the process of owned dog abandonment, as free-roaming dogs are more a symptom rather than the underlying source of future free-roaming dogs. For populations consisting of both owned and unowned dogs, where abandonment and successful breeding on the street of free-roaming dogs is large driver of population dynamics, a combination of approaches including the promotion of responsible ownership, controls on breeding or acquisition of dogs, and readily accessible sterilization services will be most effective^17^.

However, the question remains, why is CNVR sometimes successful at achieving its stated impacts and at other times reported to be insufficient^33^? What elements of CNVR are most influential on positive outcomes and what variables should be considered in its implementation to maximize effectiveness? Here, we utilize an agent-based model simulation of a generalized free roaming dog population while varying many aspects of intervention including management strategy, duration of intervention, intensity of intervention, and quality of immunization. This model builds on the work of previous models^33,34^ but expands and improves upon them by including a randomized spatial layout to avoid biases, increasing the number and type of observational data produced, and testing a wider variety of intervention methodologies.

We evaluated the efficacy of four different intervention strategies on dog population control:

- *Lethal control* in which dogs are caught and killed with no vaccination or release efforts.
- *Vaccination-only* in which dogs are caught (once in a lifetime) and vaccinated against disease with no culling or sterilization.
- *Female-only CNVR* in which only females are caught, vaccinated against disease, and sterilized while males remain untreated.
- *Male-inclusive CNVR* in which both males and females are caught and vaccinated, and females are also sterilized.

By varying duration, intensity and vaccine quality used by these intervention strategies, we were able to test four hypotheses:

1. CNVR strategies will outperform Lethal Control both for greater population reduction and longer population rebound to pre-intervention levels.
2. CNVR strategies will be associated with fewer young dogs in the population as compared to Lethal Control. Intervention that includes fertility control should prevent reproductive bursts following depressions in population density, while Lethal Control does not.
3. Longer intervention durations and higher capture rates will be associated with greater population reduction in both CNVR strategies and Lethal Control strategies as more dogs are sterilized or culled, respectively. However, because CNVR interventions reduce reproductive capacity, population reduction benefits of CNVR will be maintained for longer post-intervention than in Lethal Control for all intervention durations evaluated.
4. Vaccines that provide longer immunity will result in a higher maximum coverage in dog populations as each vaccination event will result in a longer period of protection for a given dog. Dogs that are vaccinated as part of CNVR will not be targeted for capture again, hence a reduction in the rate of transition from immune to no-longer immune is particularly important for these dogs, as this reduces the number of dogs that have outlived their immunity and become vulnerable to rabies again but will not be targeted for further intervention.

## Methods

### Model overview and description

Here we present ‘StreetDogSim’ an open-source agent-based model of an in silico dog population that matches the demographic patterns seen in vivo to assess a wide range of intervention options. Individual dogs are represented as agent with unique survival, reproduction, and other life history characteristics that either are assigned to match real-world observations or are influenced by the model’s activity. It was produced in NetLogo v6.2.2^35^ and was partially constructed using systems created previously^33,34^. A full understanding of the model’s complete mechanisms can be found in the supplementary material where they are formalized in the ODD + style^36^.

**Figure 1:**
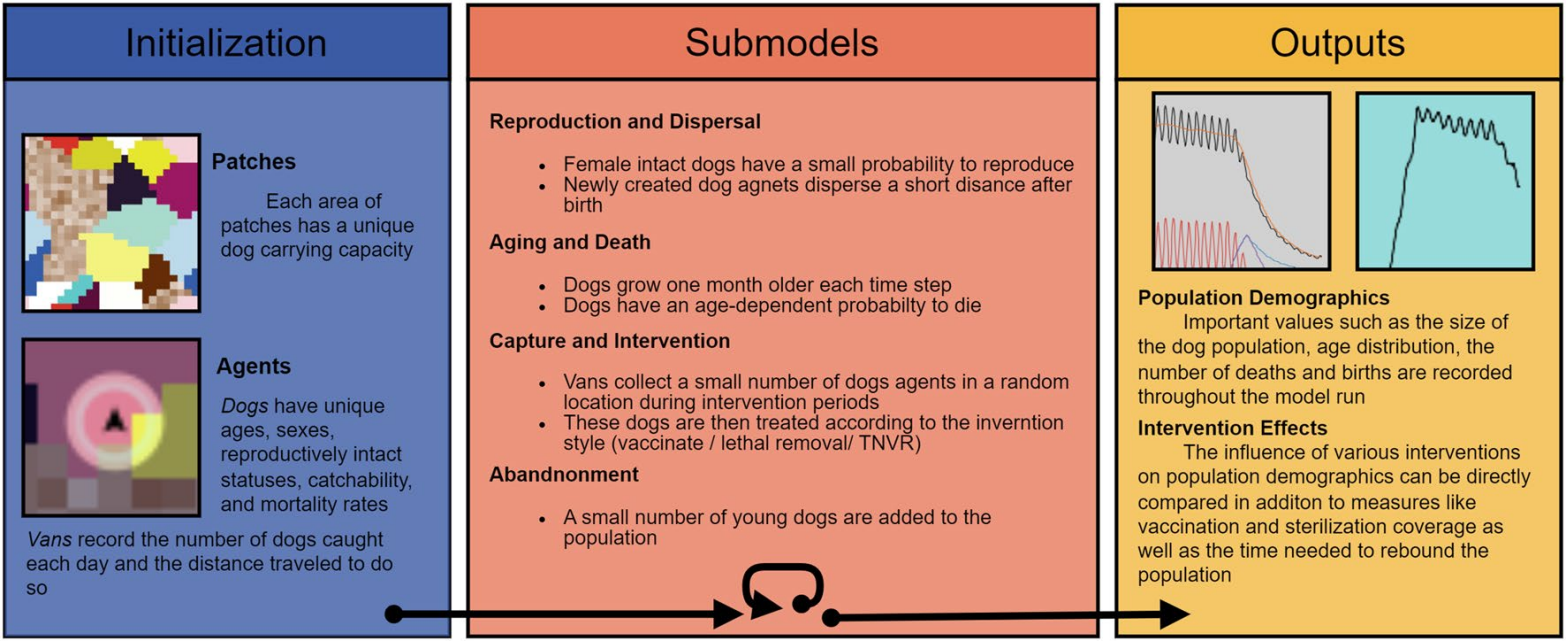
A visual representation of the model's initial setup and parameterization, the actions that are performed during the model run, and the major outputs. The environment is generated with dog and van agents that perform actions described by the submodels. Both during and at the terminus of a model run, important demographic effects are recorded and output for later investigation.

The model’s population is altered by major demographic processes (see Fig. 1), including births, natural deaths, and abandonment of owned dogs. This naturally fluctuating population is limited by the defined carrying capacity but is also altered by varied management strategy interventions (Female-only CNVR, Male-inclusive CNVR, Vaccination-only, and Lethal Control). A summary of the experimental design can be found in Table 1. The model runs for a set period of time, generally 10 years, before it is halted and model outputs are collected. This process is repeated 25 times per parameterization.

**Table 1:** An overview of the ‘standard’ model’s important parameterizations and experimental derivations from those assumptions.

Parameter	Standard Value	
Intervention used	Lethal control and female-only CNVR	
Vaccine protection period	36 months	
Intervention duration	10 years	
Capture rate	300 dogs per month	

Experiment	Parameter varied	Outcomes Compared
Intervention strategy	Lethal control Vaccine-only Female-only CNVR Male-inclusive CNVR	SPR ${T}_{SPR}$ ${P}_{SPR}$ ${\overline{n} }_{min}$ $\sum death$ ${Pup}_{\mathrm{\%}}$ Maximum vaccination coverage
Vaccine protection duration (male-inclusive CNVR only)	12 months of protection 36 months of protection	Maximum vaccination coverage Lapsed immunity percentage
Intervention DURATION	5 years of intervention 10 years of intervention 15 years of intervention	${P}_{SPR}$
Capture Rate	100 dogs captured per month 300 dogs captured per month 500 dogs captured per month	${\overline{n} }_{t10}$

### Burn in

For all models, we completed an initial 10-year burn-in in which the model ran without any intervention and these burn-in data are not included in any analyses. This was done to allow population demographics during an experimental observation period to not be influenced by the starting demographics.

### Lagging means metrics

Our model assessed population characteristics every month and calculated lagging means for the past year. We used a 12-month lagging mean rather than the value of any one individual month to mitigate the effects of any brief, extreme change in population characteristics that poorly represents average trends. For example, when a population achieves a successful reduction that means the mean population size calculated across the past 12 calendar months is at or below our defined parameter for success (see below).

### Outcomes and population metrics

The following population metrics were used as dependent variables to measure the effectiveness of population management:

*Successful Population Reduction* (SPR): A 50% reduction in the total number of individuals such that

$${\overline{n} }_{SPR}= \frac{{I}_{pop}}{2}$$

where ${I}_{pop}$ is the initial uncontrolled population represented by the lagging mean population size for the end of the last year of burn-in (before intervention) and ${\overline{n} }_{SPR}$ is the value at which that lagging mean population size is considered to be successfully reduced by half.

*Time to SPR* (${T}_{SPR}$): The number of months between intervention initiation and the month in which the population achieved ${\overline{n} }_{SPR}$.

*Permanence of SPR* (${P}_{SPR}$) Number of months after intervention ceased where population sizes stayed below 90% of the initial size, ${I}_{pop}$_._ This could also be described as a population’s ability to rebound from intervention.

*Lowest population size* (${\overline{n} }_{min}$) The lowest lagging mean number of individuals of any age in the population achieved at any time during the intervention.

*End of intervention population size* (${\overline{n} }_{t10}$): The lagging mean number of individuals of any age at the end of intervention year 10.

*Total dog deaths* $(\sum death)$: The sum of all deaths that occurred during the intervention, a proxy for dog welfare.

*Puppy percent* (${Pup}_{\%}$): The lagging mean percent of the population less than or equal to 6 months old when the intervention ends.

*Maximum vaccination coverage*: The highest lagging mean percent of the population vaccinated with functional immunity at any point during intervention.

*Lapsed immunity percentage*: The lagging mean percentage of dogs who had previously been caught and vaccinated but had outlived their rabies immunity duration at the end of the intervention.

### Intervention variables

We varied the population management interventions by four independent variables: Intervention strategy, vaccine protection duration, capture rate (thereby intensity of intervention) and duration of intervention. All statistical testing was performed using JMP v14. A sensitivity analysis^37,38^ was performed and the full results found in the supplementary material.

### Intervention strategy

We evaluated the four intervention strategies (Female-only CNVR, Male-inclusive CNVR, Vaccination-only, and Lethal Control) using a standard capture rate of 300 dogs per month, a 10-year burn-in and 10-years of intervention. Effectiveness was compared using the SPR, ${T}_{SPR}$, ${P}_{SPR}$, ${\overline{n} }_{min}$, $\sum death$, ${Pup}_{\%}$ and maximum vaccination coverage.

### Vaccine protection duration

We evaluated the effects of rabies vaccines that confer different durations of immunity by comparing periods of 12 months (what might be expected from low-quality egg culture vaccine) and 36 months (what might be expected from modern cell culture vaccine). For all immunity duration comparisons, we used the Male-inclusive CNVR management strategy with a 10-year burn-in period followed by 10 years of intervention. Effectiveness was compared using the maximum vaccination coverage and lapsed immunity percentage.

There is no epidemiological submodel here that simulates the spread of disease or impacts dog demographics. Vaccines are viewed through the lens of rabies within this manuscript as that is the reason many dog populations are managed.

### Capture rate

We compared the effect of intervention intensity by using both the Lethal Control and Female-only CNVR strategies with three different capture rates (100, 300, or 500 dogs per month). In all these scenarios, we ran models with a 10-year burn-in and 10-years of intervention with varied capture rates. Effectiveness was compared using ${\overline{n} }_{t10}$.

### Intervention duration

We assessed Lethal Control and Female-only CNVR strategies with varied intervention duration of 5, 10, and 15 years following a 10-year burn-in for all models, with capture rate controlled at 300 dogs/month. Effectiveness was compared using ${P}_{SPR}$.

### Statistics

We used ANOVAs to compare the effects of management strategies, vaccine protection duration, capture rate, and intervention duration on the previously described population metrics. ANOVAs were run separately for each of the four independent variables. For all ANOVAs $\alpha$ ≤ 0.05 was required for differences to be considered significant.

The intervention strategy ANOVA was used to assess differences in means for ${T}_{SPR}$, ${P}_{SPR}$_,_ ${\overline{n} }_{min}$, $,$ $\sum death$, and ${Pup}_{\%}$ between the four strategies (Lethal Control, Vaccine-only, Female-only CNVR, and Male-inclusive CNVR). The vaccine protection duration compared differences in maximum vaccination coverage and lapsed immunity percentage between the populations treated with vaccinations that confer 12- vs. 36-months of immunity to rabies. For the capture rate two-way ANOVAs, our independent variables were two main management strategies (Lethal Control vs. Female-only CNVR) and the capture rates (100, 300, or 500 dogs per month) and their effects on our dependent variable, ${\overline{n} }_{t10}$. Similarly, for the intervention duration two-way ANOVAs, our independent variables were the population control strategies (Lethal Control vs. Female-only CNVR) and intervention duration (5, 10, or 15 years) and their effects on our dependent variable (${P}_{SPR}$). When ANOVAs indicated significant differences between groups, Tukey’s HSD was used to assess pairwise significant differences when comparing three or more groups with $\alpha$ ≤ 0.05 indicating significance. Student’s t-test was used to assess significant when only two-groups were compared with $\alpha$ ≤ 0.05 indicating significance.

Sensitivity analyses were performed using both one-at-a-time and two-at-a-time methods to assess the effect of varying model parameters on the comparative success of Female-only CNVR and Lethal control’s ability to suppress the population size^37,38^.

## Results

### Intervention strategy

**Figure 2:**
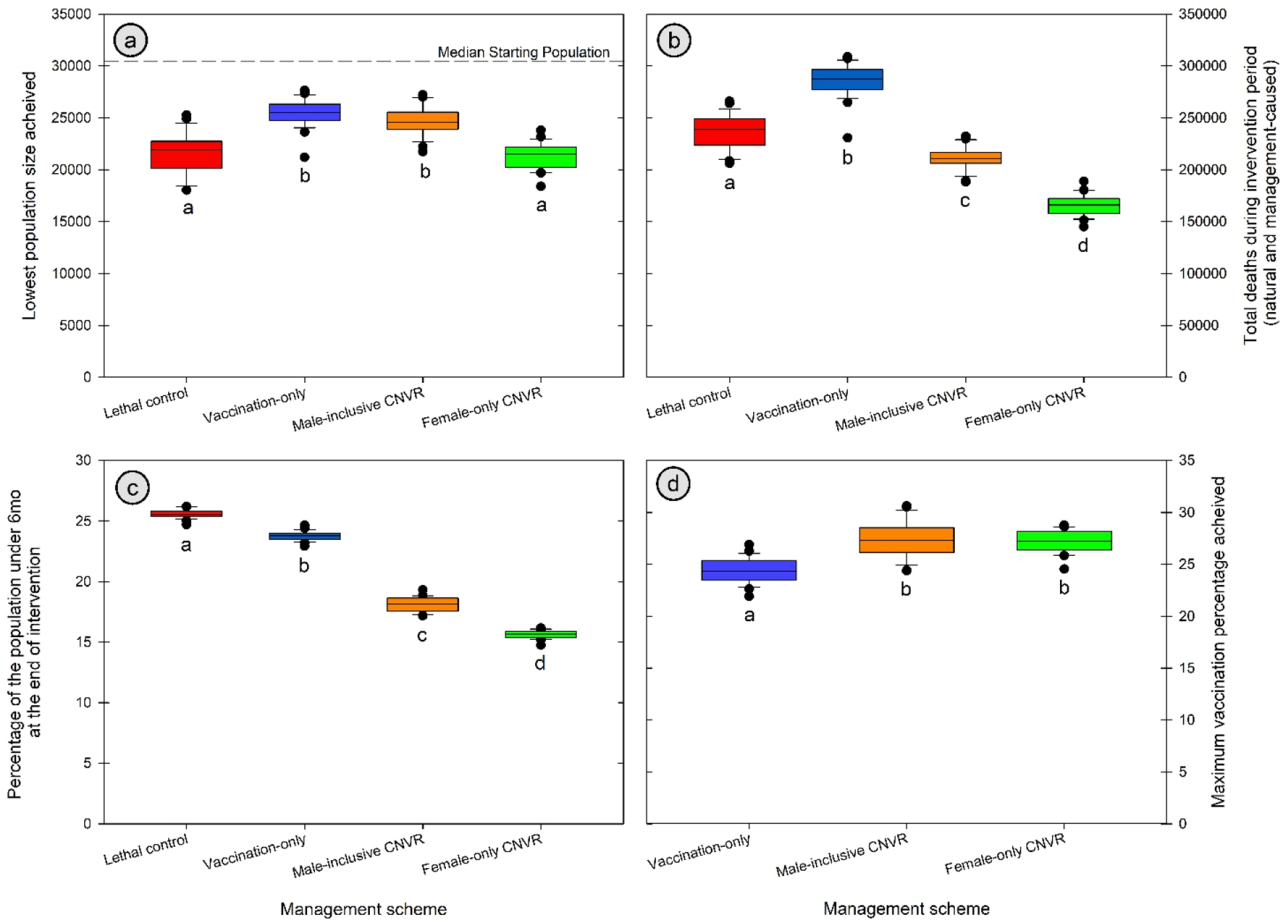
(**A**) The lowest lagging mean dog population that was achieved by the four intervention strategies, all other parameters being equivalent (300 dogs caught per month and identical population dynamics). Significant groupings are denoted by matching letters. For vaccination-only, this decline is only caused by random variation in yearly population size and is not driven by any population control. The dotted line shows a the normal population average. (**B**) The overall number of dogs that died (by natural or intervention-related means) during the 10 years of each model’s intervention period. Significant groupings are denoted by matching letters. (**C**) The percentage of the population that was under 6 months of age) at the end of each model’s 10-year intervention period. Significant groupings are denoted by matching letters. (**D**) Boxplot data shows the highest percentage of dogs that had an immunologically protective vaccination status between each relevant management strategy (all using vaccine with 36-month immunity duration). Significant groupings are denoted by matching letters for vaccination percentage. Bar data shows the mean (and standard deviation) of dog population sizes at the time the maximum vaccination percentage was achieved.

${\overline{n} }_{min}$: We found significant differences in the lowest mean population size during intervention among the four management strategies (F_3, 96_ = 49.3, *p* < 0.001, Fig. 2A) with significant differences for all pairwise comparisons except Male-inclusive CNVR / Vaccine-only and Lethal Control/Female-only CNVR (*p* < 0.001 for all significant comparisons). Female-only CNVR produced the lowest lagging mean (${\overline{n} }_{min}$ =21,302) followed by Lethal Control, Male-inclusive CNVR, and Vaccination-only control (${\overline{n} }_{min}$ =21,560, 24,594, 25,486 respectively, or a 1%, 15%, and 19% increase in lowest population size compared with Female-only CNVR).

${{\varvec{T}}}_{{\varvec{S}}{\varvec{P}}{\varvec{R}}}$**:** None of the managements scheme’s achieved the requisite 50% reduction in dog population, hence do not have a ${{\varvec{T}}}_{{\varvec{S}}{\varvec{P}}{\varvec{R}}}$ for comparison.

**Figure 3:**
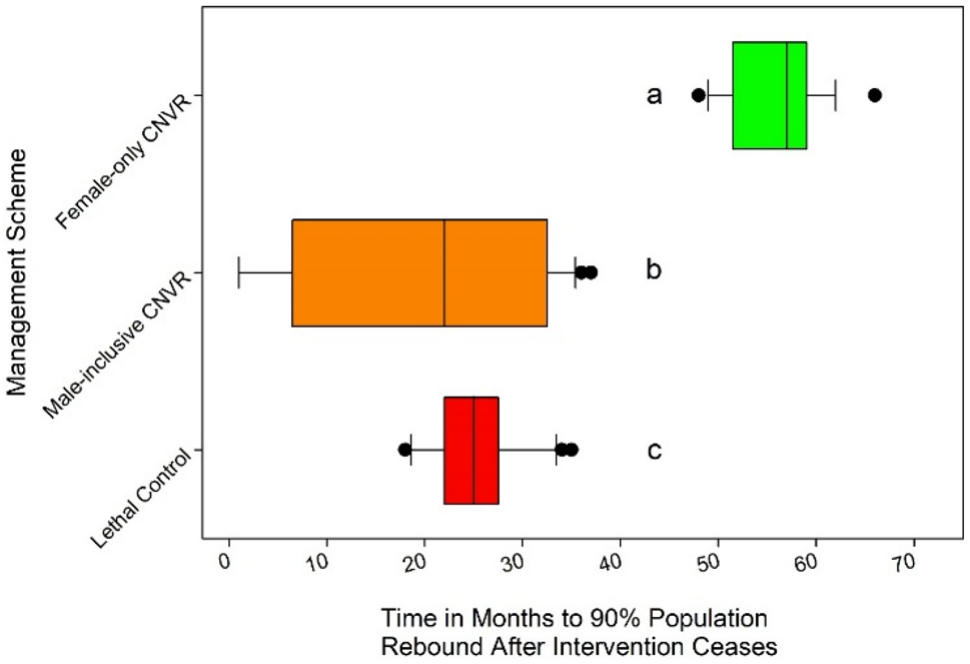
The permanence of population control by relevant management strategies as displayed by the time since intervention ceased until population numbers rebounded. Significant groupings are denoted by matching letters.

${{\varvec{P}}}_{{\varvec{S}}{\varvec{P}}{\varvec{R}}}$: The three schemes that lowered the population size (all but Vaccination-only) did so by over 10%. The time since intervention ceased for them to go over this 90% threshold for successful population management varied among groups (F_3, 96_ = 137.8, *p* < 0.001, Fig. 3). Female-only CNVR resulted in the longest ${P}_{SPR}$ of 4.66 years following the end of 10 years of intervention with 2.10 years from Lethal Control and 1.63 years from Male-inclusive CNVR. All pairwise comparisons for ${P}_{SPR}$ were significant (Tukey’s HSD, *p* < 0.001 for all but Lethal Control and Male-inclusive CNVR at *p* < 0.05).

$\sum death$ Total Dog deaths during intervention varied among the intervention strategies (F_3, 96_ = 334, p < 0.001, Fig. 2B). Female-only CNVR had the lowest $\sum death$ and was significantly lower than all strategies (total deaths = 165,343, *p* ≤ 0.001 for all comparisons). Male-inclusive CNVR had the next lowest number of deaths (210,520) followed by Lethal Control (236,823) and Vaccination-only (286,024). These strategies resulted in a significant 27%, 43% and 73% respective increase in $\sum death$ over the Female-only CNVR strategy.

${Pup}_{\%}$ The percent of the population 6 months or younger after 10 years of intervention varied significantly among all groups (F_3, 96_ = 2872.2, *p* < 0.001, Fig. 2C). All pairwise comparisons between groups were significant with Lethal Control producing the highest puppy percent followed in descending order by Vaccination-only, Male-inclusive CNVR, and Female-only CNVR (mean puppy percent 25.59, 23.76, 18.19, and 15.62, respectively, Tukey’s HSD < 0.001 for all comparisons).

*Maximum vaccination coverage* We found significant differences in the maximum vaccination coverage among the three strategies that included vaccination (F_2, 72_ = 34.6, *p* < 0.001, Fig. 2D). Female-only and Male-inclusive CNVR produced higher maximum vaccination coverage than the Vaccination-only strategy (27.19, 27.46, and 24.47 percent on average respectively, Tukey’s HSD *p* < 0.001). The difference between CNVR strategies was non-significant but both were significantly different than Vaccine-only (Tukey’s HSD *p* < 0.001).

### Vaccine protection duration

**Figure 4:**
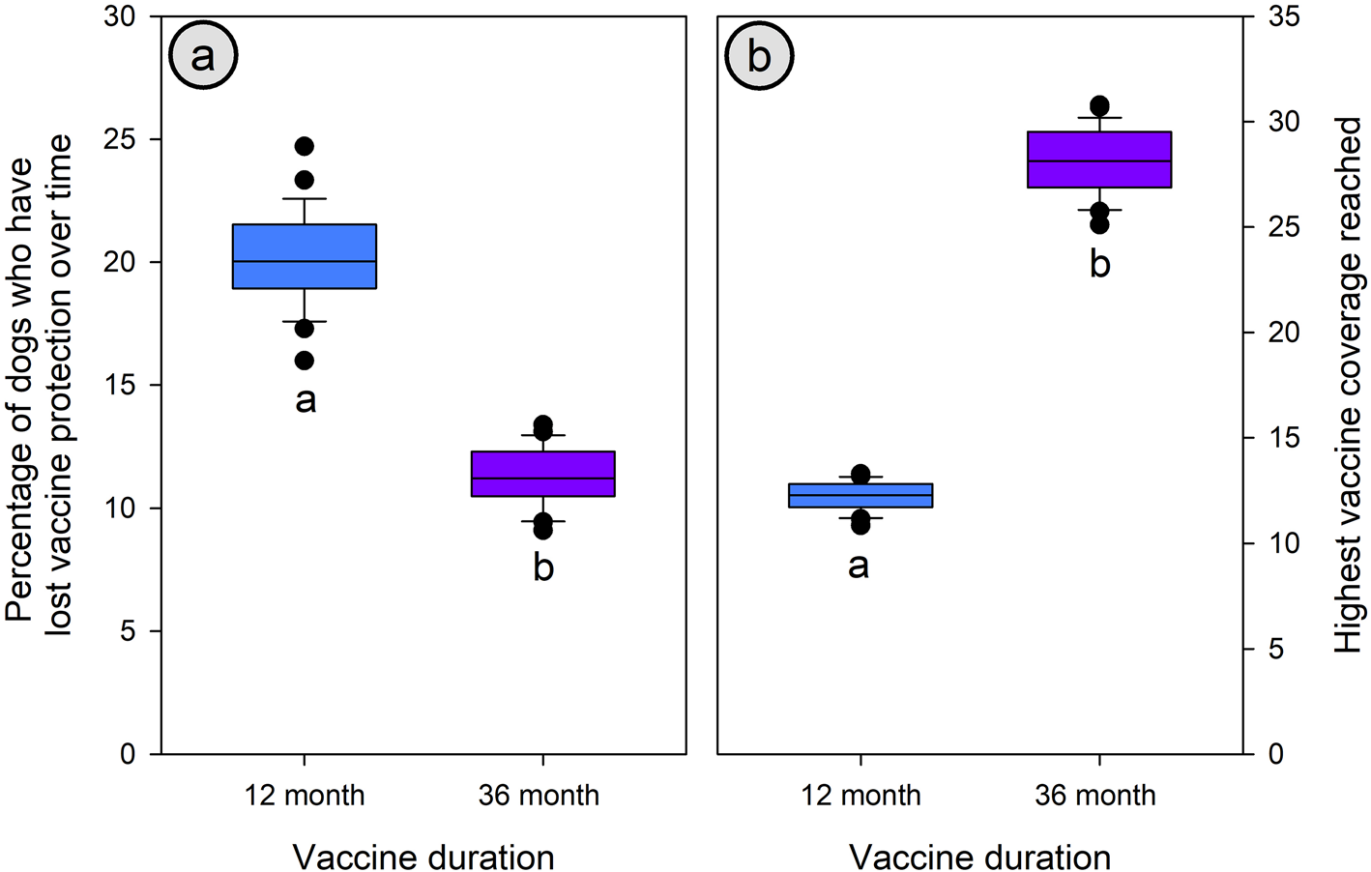
The lapsed immunity percent (percentage of the population that once had vaccine protection but had outlived it) at the end of 10 years of Male-inclusive CNVR (**A**) and the highest percentage of dogs who had an immunological protective vaccination status during the intervention (**B**). Significant groupings are denoted by matching letters.

Vaccines that confer immunity for 36 months resulted in two-times greater maximum coverage than 12-month immunity (28.13 and 12.26% respectively, Student’s t *p* < 0.001, Fig. 4) during 10 years of Male-inclusive CNVR. Similarly, lapsed immunity percent was significantly higher in the 12-month immunity duration group than in the 36-month group (20.12 and 11.31%, respectively, Student’s t *p* < 0.001).

### Capture rate

**Figure 5:**
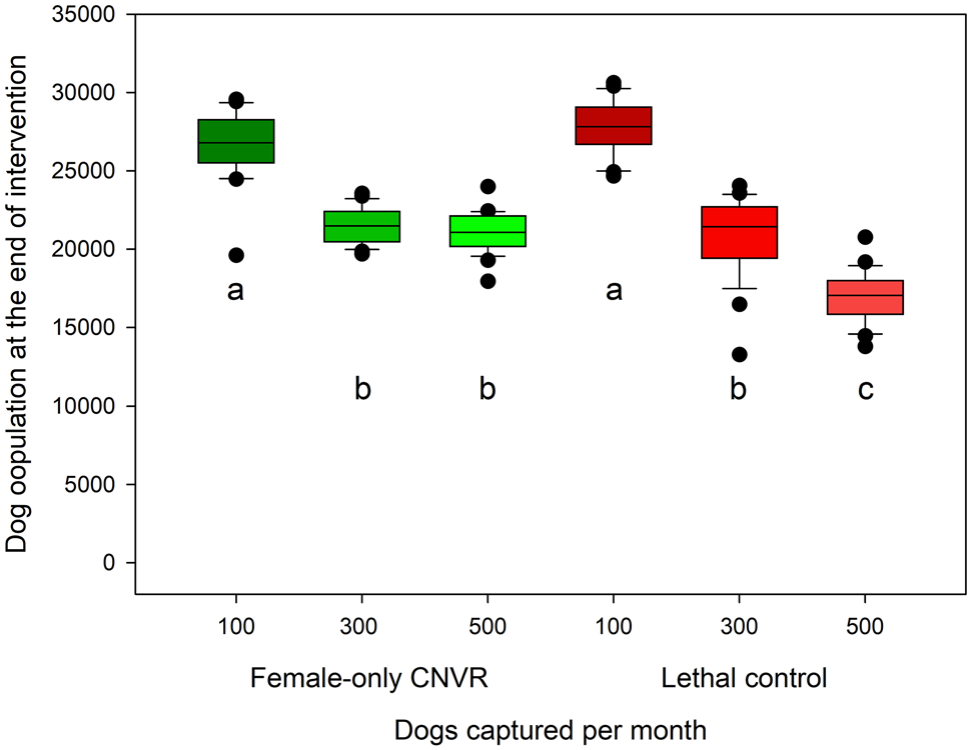
The effect of management strategy and captures per month on the mean lagging population size after 10 years of intervention. Significant groupings are denoted by matching letters.

${\overline{n} }_{t10}$: Mean lagging population size at 10 years varied significantly among capture rates (F_2, 144_ = 289.9, *p* < 0.001, Fig. 5). Capture rate was inversely related to mean population size (${\overline{n} }_{t10}$ = 27,265, 21,187, and 18,982 for capture rates of 100, 300, and 500 dogs/month, respectively when comparing across management strategies) and all pairwise comparisons were significant (Tukey’s HSD *p* < 0.001). Management strategy alone also resulted in significant differences in population size with lethal control producing a final population size of 21,852 on average, 1,252 dogs lower than Female CNVR, (t-test *p* < 0.0001). The interaction between strategy and capture rate was also significant indicating that the effects of capture rate were not uniform when applied to Female-only CNVR and Lethal Control (F_2,144_ = 28.4, *p* < 0.001, Fig. 5).

Capture rates of 100 dogs/month resulted in the highest population size in both Lethal Control and Female-only CNVR (${\overline{n} }_{t10}$= 27,803 and 26,729 dogs, respectively, Fig. 5). These groups were not significantly different from each other (Tukey’s HSD *p* > 0.05) but were significantly higher than all other capture rate by strategy combinations (Tukey’s HSD *p* < 0.001 for all comparisons). The combination of Lethal Control with capture rates of 500 dogs/month resulted in the lowest population size (16,885 dogs). The Lethal Control with 500 dogs/month capture combination was significantly lower than all other groups (Tukey’s *p* < 0.001 for all comparisons). Lethal Control and Female-only CNVR with capture rates of 300 dogs/month and Female-only CNVR with 500 dogs/month captured resulted in intermediate population sizes ranging from 20,869 to 21,505 dogs and these groups were not significantly different from each other (Tukey’s HSD *p* > 0.05, Fig. 5).

### Intervention duration

**Figure 6:**
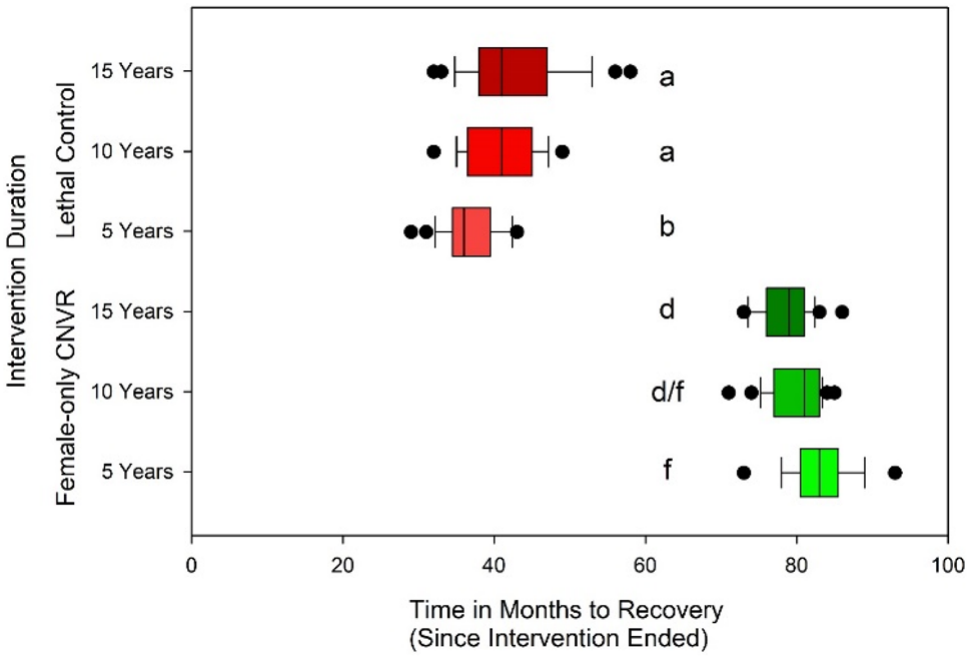
The permanence of SPR (time to rebound to 90% of initial population size after intervention ceased) by intervention strategy (at 300 dogs per month) and three intervention durations (5, 10 and 15 years). Significant groupings are denoted by matching letters.

We found significant effects of management strategy (F_1,144_ = 3070, *p* < 0.001) and its interaction with intervention duration (F_2,144_ = 18.2, *p* < 0.001) on ${P}_{SPR}$, the time to rebound to near pre-intervention population levels, but no significant effects of intervention duration alone (F_2,144_ = 0.37, *p* > 0.05, Fig. 6). The shortest intervention duration was associated with the most rapid rebound in lethal strategies however the impact of duration in Female-only CNVR was counter to the expected trends (Fig. 6). On average, 5-year interventions had a slightly shorter rebound time (4.98 years) compared with 10-year and 15-year interventions (both 5.04) and differences were non-significant. Averaging all intervention durations, the Female-only CNVR population took roughly 6.70 years after intervention cessation to rebound while Lethal Control resulted in population level rebound in just 3.35 years (Student’s t, *p* < 0.001, Fig. 6).

**Figure 7:**
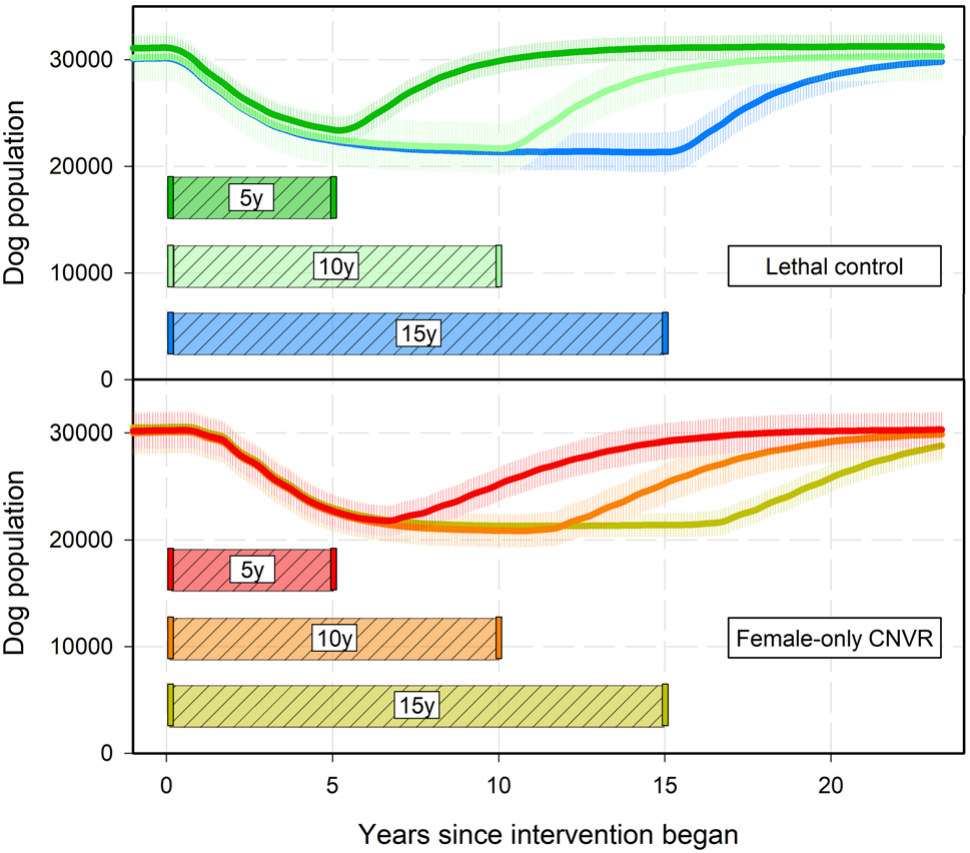
The mean dog population (and standard deviation) over time with differing management strategies (Female-only CNVR or Lethal Control) and durations (5, 10, or 15 years) at 300 dogs captured per month. Colored vertical bars signify when intervention began (0y) and ended.

We found that Female-only CNVR resulted in longer population reduction benefits than Lethal Control for all intervention durations (Tukey’s HSD p < 0.001 for all comparisons between Female-only CNVR and Lethal Control with varied intervention duration, Fig. 6). Intervention duration of 5 and 15 years were significantly different from each other in Female-only CNVR (Tukey’s HSD *p* < 0.01) but neither was significantly different from 10 years. Lethal Control intervention applied for 5 years resulted in the fastest population rebound of roughly 3.06 years (Fig. 6, Fig. 7). This ${P}_{SPR}$ was significantly shorter than the Lethal Control groups with 10 and 15 years of intervention (roughly 3.43 and 3.55 years, Tukey’s HSD *p* < 0.001 and 0.0001, respectively).

## Conclusions

Our model shows that both Lethal Control and Female-only CNVR depress population size and both strategies outperform Male-inclusive CNVR and Vaccination-only management strategies, the latter having no population size impact (Fig. 2A). However, Female-only CNVR has a much more durable impact (Fig. 3) and does so without a negative demographic shift towards a younger dogs (Fig. 2C).

The overall number of dog deaths (Fig. 2B), our closest empirical measure to ‘suffering’, shows that Lethal Control is, perhaps counterintuitively, associated with fewer dog deaths than the non-lethal Vaccine-only intervention. Depressing the population size through active killing also reduces the large number of background natural deaths that accompany large populations at carrying capacity. Boone^39^ reported the same finding from an agent-based stochastic model of cat populations; that doing nothing to manage a cat population led to more ‘preventable deaths’ than a culling strategy. Any free roaming animal management policy that is undertaken without somehow reducing the population size is a tacit acceptance of the outcome of high natural mortality. However, local communities do not give equal approval to all deaths and lethal removal (in particular when using methods such as strychnine poisoning with visibly disturbing effects) can engender distrust of local animal control programs.

The *critical* percentage of immune dogs required to eliminate rabies from a population has been estimated at 20–45%^40^. This differs from the 70% annual vaccination campaign *target*, as described by the WHO^41^ which should be sufficient to maintain the critical herd immunity in the susceptible population in the period between campaigns. As our model measures the percentage of immune dogs as a lagging mean over the previous year, rather than the number of dogs vaccinated at a particular point in time, the critical immunity coverage is a more appropriate measure of success. Here, a large pool of dogs is unavailable for vaccination, either because they are part of the 10% of the population considered ‘uncatchable’ or they are under the minimum age of six months for intervention. Additionally, a number of unvaccinated dogs are ‘abandoned’ to be free roaming and act as source for dogs needing vaccination. These abandoned dogs are aged between 0 and 1 year when abandoned, thus half of them are ‘uncatchable’ until they reach 6 months. An alternate parameterization that avoids some of these biases against effective vaccination can be found in the supplementary material with its resulting effects, though there are not large impacts on vaccination coverage. By using a vaccine that induced 36 months of immunity rather than 12 months, the maximum vaccination coverage was doubled (Fig. 4) due to a reduction in the number of dogs outliving the duration of immunity. Using a vaccine with a long duration of immunity is particularly important for real life CNVR projects where recatching previously intervened dogs for revaccination is not often used.

Including sterilization along with vaccination contributed to higher vaccination coverage compared to vaccination alone (Fig. 2) by reducing births; though this effect is statistically significant here it is unclear how large an effect it would play in the real world. Male dogs do not play any role in reproduction in this model system as they are assumed to never realistically be the limiting factor to reproduction in a free-roaming dog population.

The age demographic of the population was shifted older in CNVR interventions and younger when using Lethal Control. Female-only CNVR resulted in the lowest proportion of puppies (15.62%) and Lethal Control resulted in the highest proportion of puppies (25.59%). Lethal Control pushed the population below carrying capacity whilst retaining full reproductive potential in the (momentarily) depressed population. Puppies are a special concern in free-roaming dog populations due to both maternal aggression and rabies transmission. Reece^23^ reported a decline in dog bites in Jaipur following implementation of CNVR in part due to a reduction in population size. However, closer inspection of dog bites revealed a seasonal peak following whelping, suggesting that maternal defensive aggression motivated by perceived threat to puppies was responsible for some of the bite incidence, hence fewer puppies should be of benefit to public safety. The suffering and high mortality rate of free-roaming puppies can also be distressing for the public and lead to high levels of complaints to local authorities.

Our agent-based approach found a stronger benefit from fertility control than lethal control, in line with the same agent-based approach employed by Shamsaddini^42^, but contradicting previous system dynamics models^17,43^. Smith^17^ replicates the system in Lviv, Ukraine where ownership and sheltering play a larger role in population dynamics in contrast to the scenario replicated here where owned dogs are a smaller component of the total dog population. Lowering the influence of free-roaming breeding as a source of new individuals shifts the optimal management practices towards lethal removal by blunting the effectiveness of fertility control, as has been found in attempts to model feral cat management^30^.

Belsare and Vanak^33^ employ an agent-based approach with only a 5 year period where different intervention intensities are applied before the subsequent rebound of the population over 20 years is compared (using a similar starting population size as our model). Their “real world” scenario is perhaps closest to ours, as this includes both abandoned and uncatchable dogs. Belsare and Vanak report that high intensity (750 dogs per month) Female-only CNVR for just 5 years leads to a 20% decline in the population size that took 18 years to rebound to the initial population. Similarly, after just 5 years, with a lower intensity of 300 dogs per month, our models showed a 24.1% reduction in population by Female-only CNVR (25.4% for Lethal control and 12.5% for Male-inclusive CNVR) which rebounded around 6.75 years after intervention ceased. Belsare and Vanak describe their outcome as ‘not effective’, however a 20% decline in the population size and 18 years of population suppression could feasibly be perceived as a success, particularly if Lethal Control is considered an especially unpalatable alternative.

We tested a wide range (up to 15 years) of intervention durations, as compared to the 5^33^ or 10 years^42^ of others. Although 5 years of funding is not unusual for DPM, these management systems should provide permanent community services^15^. Ongoing management is reflected in Smith^17^ using 70 years for their intervention model runs and in Larkins^27^ analysis of 23 years of data on the rabies prevention cost effectiveness from a real-world ongoing CNVR intervention in Jaipur, India. Our experiments which vary the duration of the intervention period show that increasing durations leads alterations in the speed of population rebounding (Fig. 6), though this trend is unexpectedly reversed for CNVR. It is unclear why this might have occurred and bears further study. These differences demonstrate the importance of using accurate local parameters, the sort of flexibility our model is designed for. Belsare and Vanak^33^ simulate the efforts required to capture dogs by stochastically varying the number captured per time period and applying this consistently throughout the model run. Here we mimic the real-world trend of increasing difficulty of capturing new dogs as the population is saturated with already intervened dogs by dynamically limiting captures per time period using an effort limiting effect driven by the maximum distance that can be traveled each day looking for new dogs (see Supplementary Material for full details).

This model is an extension of prior work^34^ and finds a similar degree of benefit of fertility control over lethal removal, however with the introduction of male-targeted control, randomized cityscapes, uncatchable dogs, vaccine duration, and effort-limiting intervention we produce a more representative system and give future users greater control over important model parameters. The introduction of waning immunity here highlights the significance of those *lapsed immunity dogs* who appear vaccinated but have lost their vaccine protection. For example, when using low-quality 12-month vaccine, these lapsed immunity dogs outnumber those with current vaccine protection by over 2:1 (Fig. 4).

We recognize that implementing sterilization in CNVR interventions in addition to vaccination has significant financial implications. CNVR may also be more costly on a per-capita basis than Lethal Control, although Benka^44^ found lethal control to be more costly than CNVR of free-roaming cats in the US. The increased costs of sterilization in addition to vaccination (incurred by the DPM implementer) may be balanced by reduced costs (saved by the public and health care) of bite treatment driven by the reduction of puppies and related maternal defensive aggression^23^, and the reduction in rabies treatment and DALYS^27^ due to greater maximum vaccination coverage, but such cost-benefits analysis is beyond the scope of our model.

CNVR implementation methods can potentially maximize cost effectiveness. Benka^44^ found their free-roaming cat population model predicted ‘front-loading’ (implement most of the sterilizations in the early period of the project followed by a lower level of maintenance) CNVR sterilization effort leads to far greater population reductions than spreading the same number of sterilizations evenly across time. A previously published version of this model predicted that an annual survey of streets to identify the location of un-intervened females could help target CNVR efforts where they were most needed, minimizing the travel costs involved in dog catching and leading to lower population sizes and fewer puppies for the same intervention efforts^34^.

Of the intervention strategies investigated here, Female-Only CNVR clearly produces the most desirable population with the required elements of a smaller, healthier population vaccinated against zoonotic disease. We propose the impact of CNVR can also be enhanced with higher intensity, longer duration and the use of a quality vaccine that confers 36 months of immunity against rabies. Lower free-roaming dog population densities and turnover are a bridging force towards reducing the demonstrably negative aspects of free roaming dogs and can be combined with services and regulations supporting more responsible and humane dog ownership^14,15^.

### Supplementary Information

Supplementary Information 1.

Supplementary Information 2.

## Data Availability

This model is in the process of being published with the ComSES model repository and will be publicly available. It will also include all the data used for this manuscript.
