# Supplementary material for: Assessing multiple free-roaming dog control strategies in a flexible agent-based model
Source: Sci Rep. 2023 Nov 14;13:19826. doi: 10.1038/s41598-023-47076-x (PMC10645819; doi:10.1038/s41598-023-47076-x)
Supplement: Supplementary file 1 — Supplementary Information 1. [file 41598_2023_47076_MOESM1_ESM.docx]

**Supplementary Materials**

**ODD+2**

**Model Description**

This model was created in the agent-based modeling software NetLogo (v6.2.2, ^1^ and can be completely re-created using this standardized ODD+ document ^2^. A randomized cityscape is created and populated with ‘dogs’ who live simulated lives that mimic important processes in free roaming dogs’ life histories. Agents acting as ‘dogcatchers’ attempt to collect dogs for intervention (using either vaccination, lethal removal, sterilization, or some combination).

**Purpose**

The model seeks to provide a highly flexible platform to test different methodologies of dog population management while ensuring the outcomes match real world scenarios as closely as possible. A previous model iteration by Yoak^3^ as well as a similar model by Belsare and Vanak^4^ form the basis for strong, biologically accurate foundation but have been enhanced here for ease of use and to handle more variable situations.

**Entities, state variables, and scales,**

The model consists of two entities: dogs and dogcatchers and each has their own unique parameters (Supplementary Table 1. Agent and environmental parameters. Dogs possess nearly all of the important state variables in the system such as location, sex, age, if they are reproductively intact, if they have been previously caught (i.e., tipped, as a common marking technique is to remove a small portion of one ear tip or create a recognizable ‘notch’ in the leading edge of one ear), and if they are currently protected immunologically by a vaccine.

Dogcatchers have a location and a limit to the number of dogs they can capture that month. Dogcatchers are also limited in how much ‘effort’ they can employ by limiting their distance travelled per month. Square patches of land represent approximately 1km x 1km space (however scale has little impact on the model and will vary as users produce their own city-maps) and are stochastically broken up into zones of variable size and dog carrying capacity during model initialization. Each time step represents one month.

**Supplementary Table 1.** Agent and environmental parameters

| Entity | Parameter | Meaning | Default Value or Range | References |
| --- | --- | --- | --- | --- |
| Dog Agent | Sex | Male or Female | - | - |
|  | Age | In Months | - | - |
|  | Sterilization Status | Sexually intact or not | - | - |
|  | Location | XY coordinates | - | - |
|  | Vaccination Status | In the window of time where they have protection from a vaccine (either 12 or 36 months) | - | - |
|  | Previously Caught Status | Have they been ‘tipped’, that is previously caught be a capture van | - | - |
|  | Catchable Status | Is this dog able to be targeted by a capture van for intervention? | - | - |
|  | Monthly Adult Mortality Probability | Probability of dying for dogs Over 1 year old | $1-0{.75}^{1/{12}}$ | Derived from ^5^ |
|  | Monthly Puppy Mortality Probability | Probability of dying for dogs Under 1 year old | $1-0{.35}^{1/{12}}$ | Derived from ^3,5^ |
|  | Litter Size | Average Number of puppies produced per reproduction | 5.62 | ^6^ |
|  | Reproductive Rate | The probability that a dog will reproduce | See Supplementary Table 2 | Derived from ^6^ |
| Dogcatcher Agent | Location | The randomly decided XY coordinates | - | - |
|  | Capture Limit | The maximum number of dogs that can be caught each moth | Varies by model from 100-300 | - |
|  | Distance Limit | The maximum distance that can be traveled to catch dogs each month | See Intervention Effort Limiting Submodel description | - |


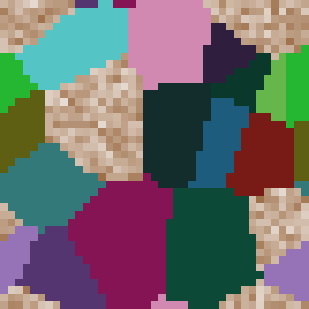

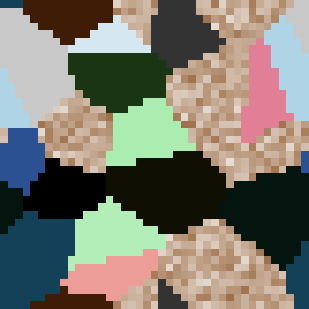


**Supplementary Figure 1**. Two possible spatial distributions of generic city zones (solid colored areas) and outside areas which allow no dog populations to persist there (brown patchy areas).

**Process overview and scheduling**

Each month, a series of dog demographic and intervention submodels are applied to the whole population in a consistent fashion. First, dogs (female, sexually intact, sexually mature) can reproduce given certain constraints determined by the reproduction submodel. Next, all dogs grow older and have some age-dependent probability of mortality during the aging submodel. Then, any intervention actions that are taking place are handled by the dogcatcher actions submodels. Finally, a small number of young, intact dogs are generated by the abandonment submodel.

**Design Concepts**

*Basic Principles*

This system is heavily influenced by population dynamics in carrying capacity limited populations and focuses on the interplay of vacuums forming (through natural death or lethal removal by dogcatchers) and filling in (with compensatory reproduction). A model that accurately reflects the real world situation is highly beneficial to free roaming dog control ^3,4,7,8^.

*Emergence*

Our primary results, such as the total dog population or the vaccine coverage, emerge from the interplay between the intervention-focused submodels (sterilization, lethal removal, or vaccination) and the dog demographic submodels (reproduction, deaths, and abandonment).

*Adaptation*

Dog reproduction changes based on the surrounding local conditions. It is the normal rate at high local population densities but progressively at lower city densities. Dog mortality rates progressively increase as the carrying capacity is approached. The number of dogs that are caught by the dogcatchers is lowered by the effort-capping submodel because as the system contains fewer dogs (in both fertility and lethal control) or fewer dogs that were not previously caught (in fertility control) it becomes harder to find a new dog.

*Objectives*

Dogs have no in-built objectives other than to reproduce and survive, and no way to influence events. Dogcatchers nominally seek to reduce the dog population and/or increase the proportion of dogs who are vaccinated but, other than how many dogs were caught each month and what they do with a dog in hand, have no actionable decisions.

*Interaction*

Dogs express an limit on survival due to crowding. Dogcatchers ‘catch’ a dog and the relationship is determined by the model run’s management strategy (i.e., kill it, vaccinate it, sterilize it).

*Stochasticity*

All probability-based actions, including dog reproduction and mortality, where dogcatchers travel to, and initial seeding of the area are random effects.

*Collectives*

There are no collective agent groups, but patches are assigned to be part of a block of patches in a city zone during initialization. Each zone patch has the same dog carrying capacity as all other patches in the same zone.

*Observation*

Many demographics factors are collected each time step about dogs:

- Age
- Reproductive status
- Immunization status
- If they had previously been caught
- If they are catchable
- XY Location

And were collated into population-level indices:

- Number of dogs
- Number of vaccinated dogs
- Number of puppies

No information is hidden from observation and perfect knowledge of all indices is assumed for the system.

*Initialization*

The cityscape is created using the map randomization submodel to produce a novel city zone shape each model run but keeps the overall carrying capacity of the system the same. Then each patch creates a few dogs equal to 90% of its’ individual per-patch carrying capacity and randomly assigns them demographic variables:

Reproductively Intact – Yes.

Vaccination Status – None.

Difficulty – In the standard model 10% are considered ‘uncatchable’ and cannot be targeted by intervention.

Sex – 50% probability to be assigned male at initialization (50% female)

Age – Assigned an age in months by randomly pulling a value from an exponential distribution of ages with a mean of 2 years (**Supplementary Figure 2**).


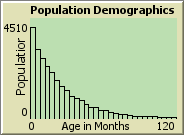


**Supplementary Figure 2**. A sample model initialization of ages

Every model run begins with 10 years of ‘burn-in’ time where the model runs without intervention to remove any effects of the initial model’s characteristics.

**Input Data**

The model does not use input data to represent any known processes.

**Submodels**

Generic *Map Randomization*

The map in the standard model is a 25x25 patches in size area, but

20 city zones are randomly generated by placing 20 placeholder agents at random XY coordinates. They then assign all the patches that are closest to them to be part of a zone which is assigned an equivalent dog carrying capacity. This per-patch carrying capacity figure is produced by pulling from a normal distribution with a mean equal to the target carrying capacity per km (30 in this model) and a standard deviation of 20% of the mean (30 * .2 = 6 here) to produce some degree of between-zone variation. 5 of these zones have their carrying capacity reduced to 0 to simulate areas out of the city or otherwise inaccessible to dogs.

When the 15 remaining zones are all assigned their carrying capacity, they are checked to ensure that the city as a whole still has a carrying capacity roughly equal to the target carrying capacity (30 dogs per patch here). If, say, one or more large zones had an exceptionally high (or low) randomly assigned carrying capacity then the city would be able to support an abnormally large (or small) dog population, and this would make between-run comparisons fraught. To avoid this, the final step of map randomization is to subtly lower a random zones per-patch carrying capacity by 1 and repeat this process until the city-wide average is only 10% above from the target city-wide average (this would increase a zone’s carrying capacity in the event the city-wide average is more than 10% lower than the target).

*Real Word Map Randomization*

If the user chooses, they can insert a outline of a real world cityscape in black and have the model populate it with variable density zones and dogs identically to how a generic map is made (Supplementary Figure 3).


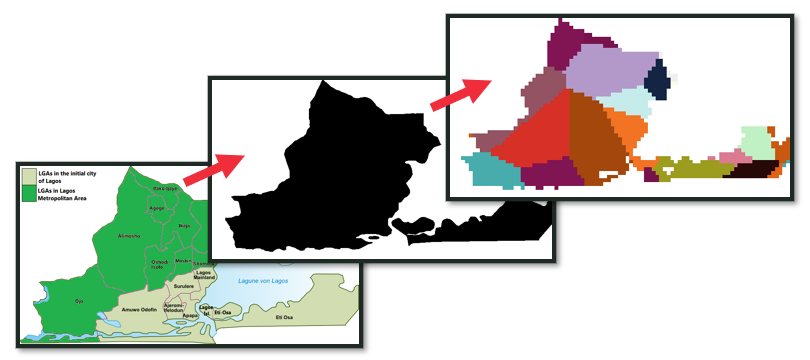


Supplementary Figure 3. Taking a real-world location and producing a outline of the city, the model can populate that area with spontaneous zones.

*Dog Breeding*

Female, intact dogs over the age of 12 months have a monthly probability to reproduce that follows the seasonality of Jaipur, India’s street dog population (in the base model, but this can be adjusted easily with sliders). The number of female dogs who breed in a given year is set by the user (47.5% here) and then the model distributes these reproductive events throughout the year based on:

1. The lagging mean population of intact, adult female dogs
2. The monthly ratio of reproductions that take place in the current month
3. The ratio of the current dog population vs the citywide carrying capacity.

If a female reproduces, she creates a litter with a size produced by rounding a normal distribution with a mean of 5.62 and a standard deviation of 1.0. Newborn puppies are randomly assigned male or female, born with no vaccination regardless of the mother’s status, and disperse five patches away in a random direction. Some portion of dogs are assigned a status of ‘uncatchable’ at birth for their entire lifespan and this makes them wholly invisible to the dogcatchers.

| **Month** | **Probability That Any Given Reproductive Event Occurs in That Month** | **Relative Likelihood to Occur in That Month Compared to the Least Likely Month (Apr)** |
| --- | --- | --- |
| Jan | 8.31 | 19.8 |
| Feb | 3.53 | 8.4 |
| Mar | 1.26 | 3 |
| Apr | 0.42 | 1 |
| May | 0.59 | 1.4 |
| June | 1.76 | 4.2 |
| July | 4.53 | 10.8 |
| Aug | 9.57 | 22.8 |
| Sept | 15.53 | 37 |
| Oct | 19.82 | 47.2 |
| Nov | 19.73 | 47 |
| Dec | 14.95 | 35.6 |

**Supplementary Table 2.** The distribution of reproductive events across a year, presented as either a percentage of the total or a relative ratio to the least month that is least likely to have a reproductive event occur in it (April).

If the population as a whole is pushed substantially below its’ carrying capacity, the model simulates the compensatory reproduction effects by using the following table.

| Current Dog Population : Carrying Capacity Ratio | Effect on Reproduction |
| --- | --- |
| >=0.9 | 0 |
| <.9 and >=0.8 | +2% |
| <.8 and >=0.7 | +4% |
| <.7 and >=0.6 | +6% |
| <.6 and >=0.5 | +8% |
| <.5 and >=0.4 | +10% |
| <.4 and >=0.3 | +12% |
| <.3 and >=0.2 | +14% |
| <.2 and >=0.1 | +16% |
| <.1 | +18% |

Supplementary Table 1. The compensatory reproduction rate increases on reproduction

*Dog Mortality*

Each month, dogs have a probability to die determined by their age class, either older or younger than 1 year. Younger dogs have a monthly probability that corresponds with a yearly survival rate of 35% and older dogs’ survival rate corresponds to a yearly rate of 75%. This matches approximately the observed mortality in the real world ^5^ and has been previously used in prior modeling efforts ^3^.

However, in this model, a dog’s chances of survival decreases as the patch it is on approaches its unique carrying capacity. For example, Adults follow this formula in patches at <75% of carrying capacity:

$$Monthly Mortality Probability= {1-0.75}^{\left( \frac{1}{12} \right)}$$

$$Monthly Mortality Probability= 0.024$$

$$Monthly Mortality Probability= 2.4\% ofAdult dogs will die$$

And this formula in patches with >= 75% of their capacity filled:

$$Monthly Mortality Probability= \left[ {1-0.75}^{(\frac{1}{12})} \right]+\left[ 1-{((0.75}^{\left( \frac{1}{12} \right)})*\frac{Count Other Dogs on Patch}{Patch Carrying Capacity} \right]$$

For example, if there are 120 dogs on a patch that should be supporting 100, the monthly probability of death for an adult dog would be:

$$Monthly Mortality Probability= \left[ {1-0.75}^{(\frac{1}{12})} \right]+\left[ 1-{((0.75}^{\left( \frac{1}{12} \right)})*\frac{119}{100} \right]$$

$$Monthly Mortality Probability= \left[ 0.024 \right]+\left[ 0.024*\frac{119}{100} \right]$$

$$Monthly Mortality Probability= \left[ 0.024 \right]+\left[ 0.028 \right]$$

$$Monthly Mortality Probability= 5.17\% of dogs will die$$

*Abandonment*

Each month, dogs are abandoned to the street by previous owners. To accommodate flexible model structures, this is assumed to be 10% of the starting stable population size at the end of the ‘burn in’ period and this 10% is spread out over 12 months. During burn-in, this added dog number is assumed to be 10% of the average over the last 12 month’s population sizes. After burn in, this number no longer changes and is ‘locked in’ as the starting population size. Newly abandoned dogs are assumed to be unvaccinated, randomly assigned either sex, have a 10% chance of being uncatchable, and aged from 0 to 12 months old.

*Intervention* *Effort Limiting*

When the model begins intervention, it has a target number of dogs to capture each month and what intervention strategy will be applied to them. To account for the increase in effort that goes into finding new dogs in a city increasingly filled with already vaccinated and sterilized individuals (in the vaccine or fertility control-based strategies) or in a city increasingly depopulated (in the lethal or fertility control-based strategies), we begin to limit how far a dogcatcher can travel each day after a 3-year training period.

For the first 3 years of intervention a dogcatcher agent is created at a random location each month, then turns to the nearest catchable dog (one that is not part of the ‘uncatchable’ dogs, nor has been previously caught). It travels the distance to that dog then turns to face a new catchable dog. The sum of the distance required to travel to catch the target number of dogs is recorded for each month. The 10% highest distances are recorded, and the next highest distance is now the maximum allowable driving distance (Supplementary Figure 2). After this ‘training period’, the dogcatcher still moves towards dogs and records distance in the same way, but now when the maximum allowable driving distance is met, the month’s intervention is finished irrespective of whether it met its target goal or not (Supplementary Figure 5).


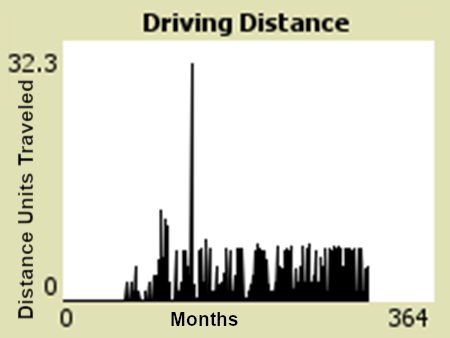


**Supplementary Figure 4.** A sample recording of the distances required to find the 300 target dogs in this model. Note the distances are capped after the initial 3-year training period.


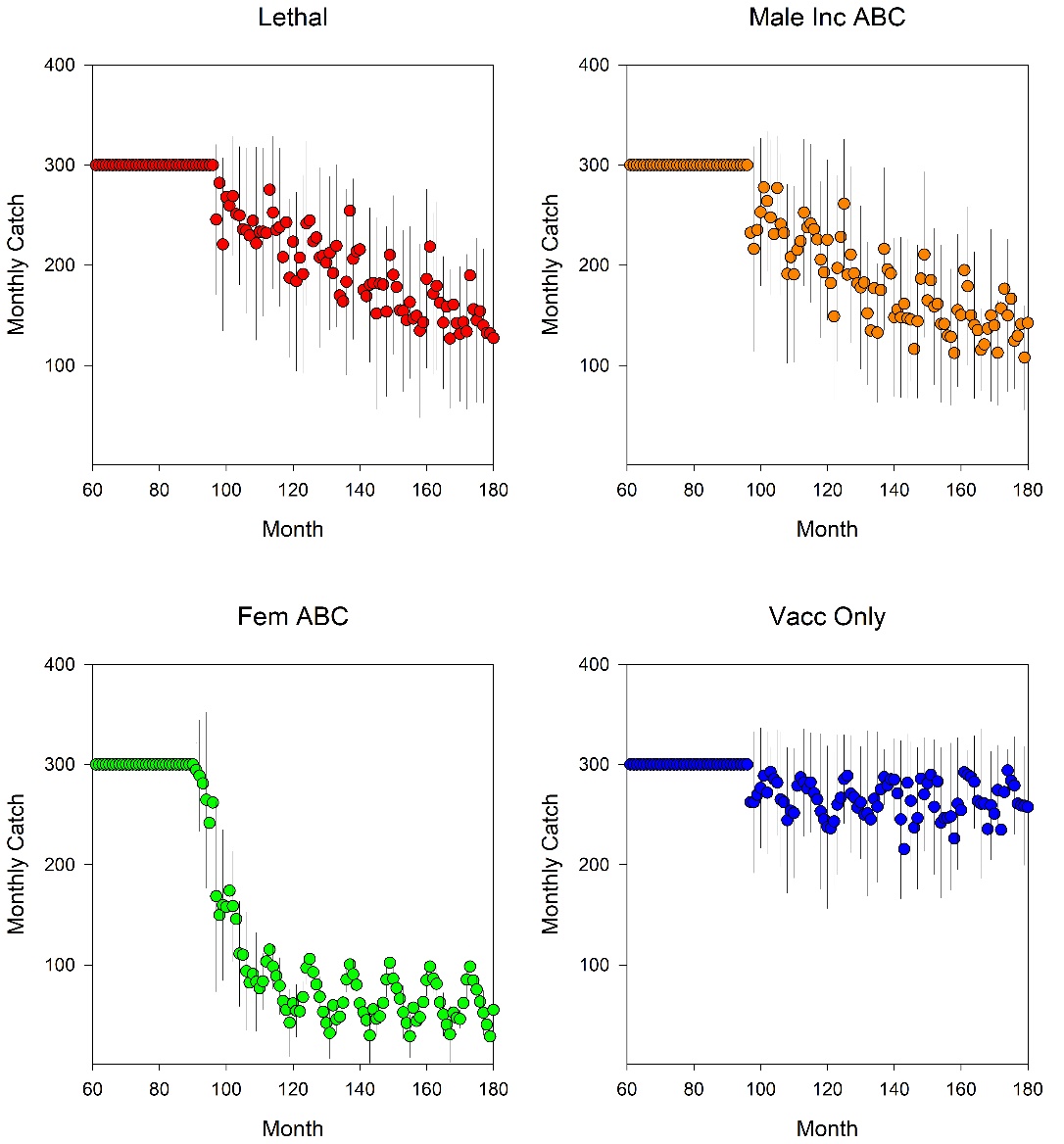


**Supplementary Figure 5.** The number of dogs that were caught each month is continuous at 300 dogs per month for the first 3 years, then is reduced as the effort cap begins to be limiting.

*Intervention Application*

As a dogcatcher selects the closest catchable dog (over 6 months, not previously caught, sex-determined by management strategy), it alters the dog’s status and increases the total number of interventions that were performed. This alteration can come in several forms, from killing the dog agent and removing it from the system (as in lethal control), or by sterilizing and/or vaccinating it (as in CNVR or vaccination strategies).

**Pattern Oriented Model Parameterization**

Models are not reality, but the approximation should behave as similar as possible under the narrow circumstances of the effect under investigation. We have made every attempt at generating a model with specific parameterizations that produce outputs with the same patterns as previously observed in the real world.

To test these effects in our model, we utilized a generic parameterization of:

- 15,000 carrying capacity
- 150 female dogs sterilized per month
- 75% adult survival
- 35% juvenile survival
- 10% abandonment
- 10% uncatchable
- Or referred back to the main paper’s data.

| Pattern | Source | Street Dog Sim | Quality of Pattern Match |
| --- | --- | --- | --- |
| Age | 14.5% Juvenile Dog (<1yo) percentage without significant management ^9^ | ~32-41% Sub Adult (<12m) variation throughout the year prior to management (Supplementary Figure 6  ~8-35% Juvenile dog (<6m) variation throughout the year prior to management (Supplementary Figure 7) | Moderate |
|  | ~4-27% Puppy (<12w) percentage varying throughout the year prior to intervention^10^ | 2-26% Puppy (<3m) variation throughout the year before intervention (  Supplementary Figure 8) | Excellent |
|  | ~1-12% Puppy (<12w) percentage varying throughout the year after intervention^10^ | 2-15% puppy (<3m) variation throughout the year after intervention (Supplementary Figure 10) | Excellent |
| Population Change due to Female Only CNVR Intervention | 15% Decline over 2 years^10^ | ~5% decline over 2 years (Supplementary Figure 11) | Poor |
|  | 30% Decline over 6 years^11^ | ~27% decline over 6 years (Supplementary Figure 11) | Excellent |
| Intervention Related | 60% of females in a population are neutered after 4y ^11^ | ~54% of females are neutered after 4y (Supplementary Figure 12) | Excellent |
|  | 75% of population return to pre-lethal control intervention levels after 8m | Slower return to pre-lethal levels, generally 30-45m (main paper data) | Poor |

Supplementary Table 2. A comparison of this models’ major outputs and observations found in the real world.


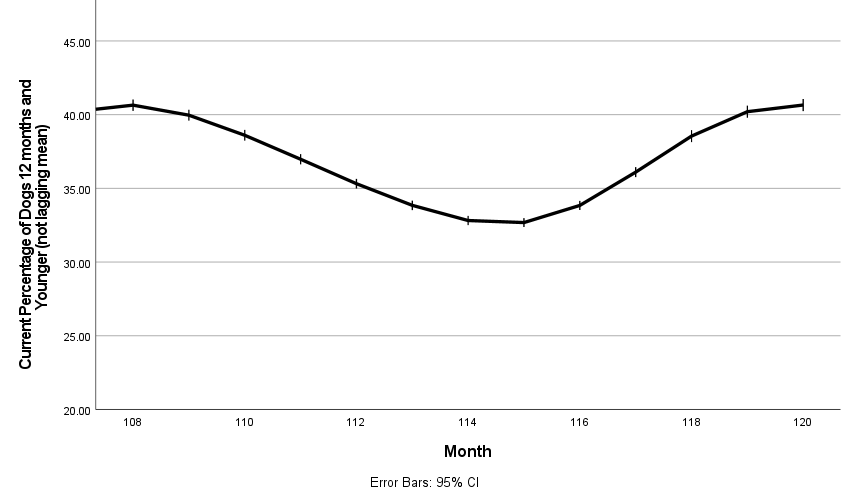


Supplementary Figure 6. The percentage of dogs that are under the age of 12 months in a generic parameterization of a pre-intervention population.


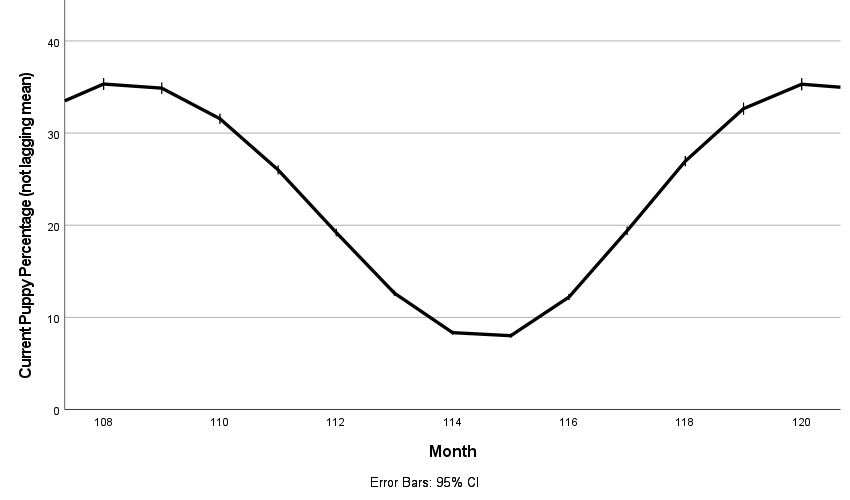


Supplementary Figure 7. The percentage of dogs that are under the age of 6 months in a generic parameterization of a pre-intervention population.


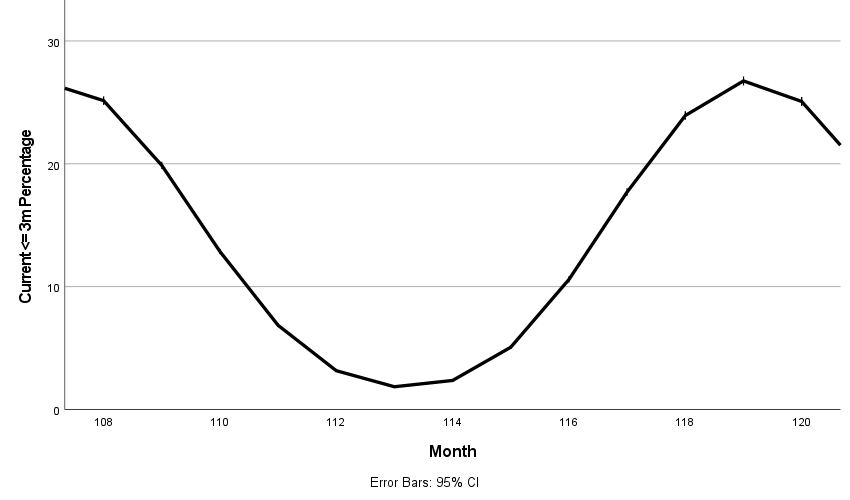


Supplementary Figure 8. The percentage of dogs that are under the age of 3 months in a generic parameterization of a pre-intervention population.


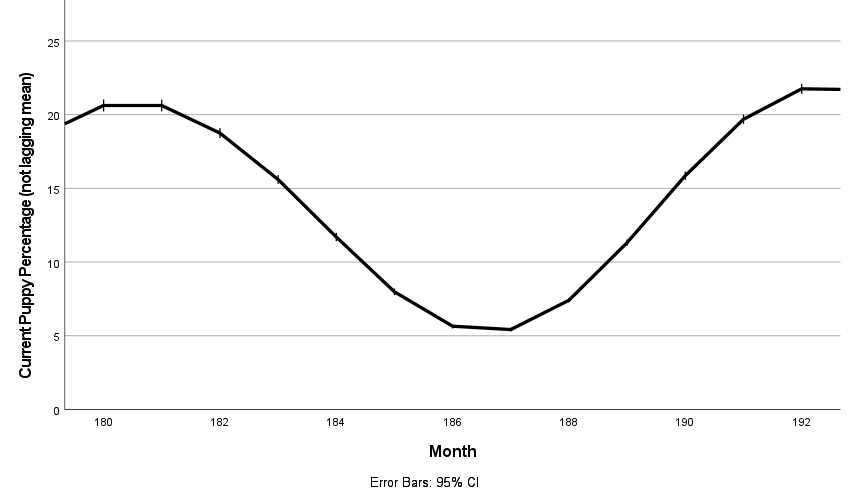


Supplementary Figure 9. The percentage of dogs that are under the age of 6 months (puppies here) in a generic parameterization of a post-intervention population.


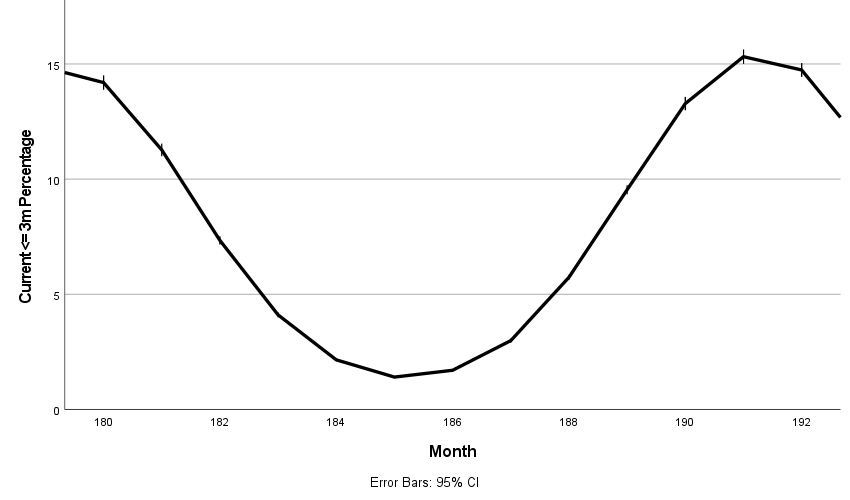


Supplementary Figure 10. The percentage of dogs that are under the age of 3 months (puppies here) in a generic parameterization of a post-intervention population.


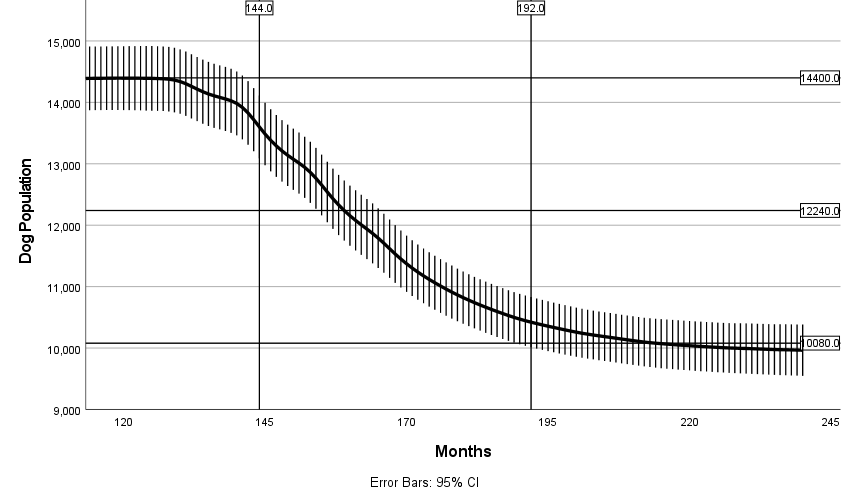


Supplementary Figure 11. The decline in the dog population size over time for a generic parameterization of female only CNVR. 12,240 represents a 15% reduction and 10,080 represents at 30% reduction.


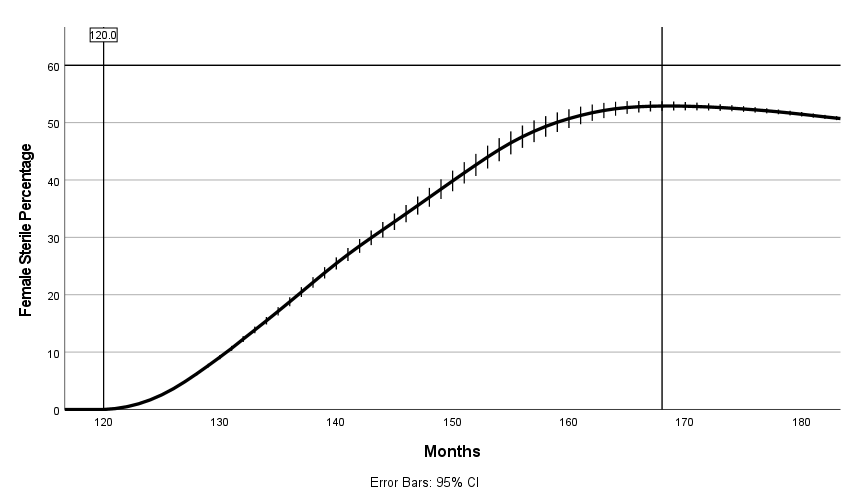


Supplementary Figure 12. The rate of increase in the lagging yearly mean female sterilization percentage over time for a generic parameterization female only CNVR.

**Sensitivity Analysis**

Since this model’s parameterization will invariably have an influence on the outcomes and not all local dog population’s population dynamics match our ‘standard’ model here, we varied the important parameters that drive population dynamics (uncatchable percentage, abandonment rate, juvenile survival, adult survival, and reproductive rate) and investigated the final dog population achieved at the end of 10 years of intervention . This was done for both Lethal Control and Female-only CNVR after which a one-way ANOVA with Bonferroni correction was used to investigate if there were significant differences between scheme treatments.

Uncatchable percentage and abandonment rate are both 10% in the standard model and we test variations from 0 to 10% here (Supplementary Figure 13). Survival was increased and decreased by 2% and 4% (Supplementary Figure 14). Reproductive rate was increased and decreased by 2.5%, 5%, and 7.5% (Supplementary Figure 15).


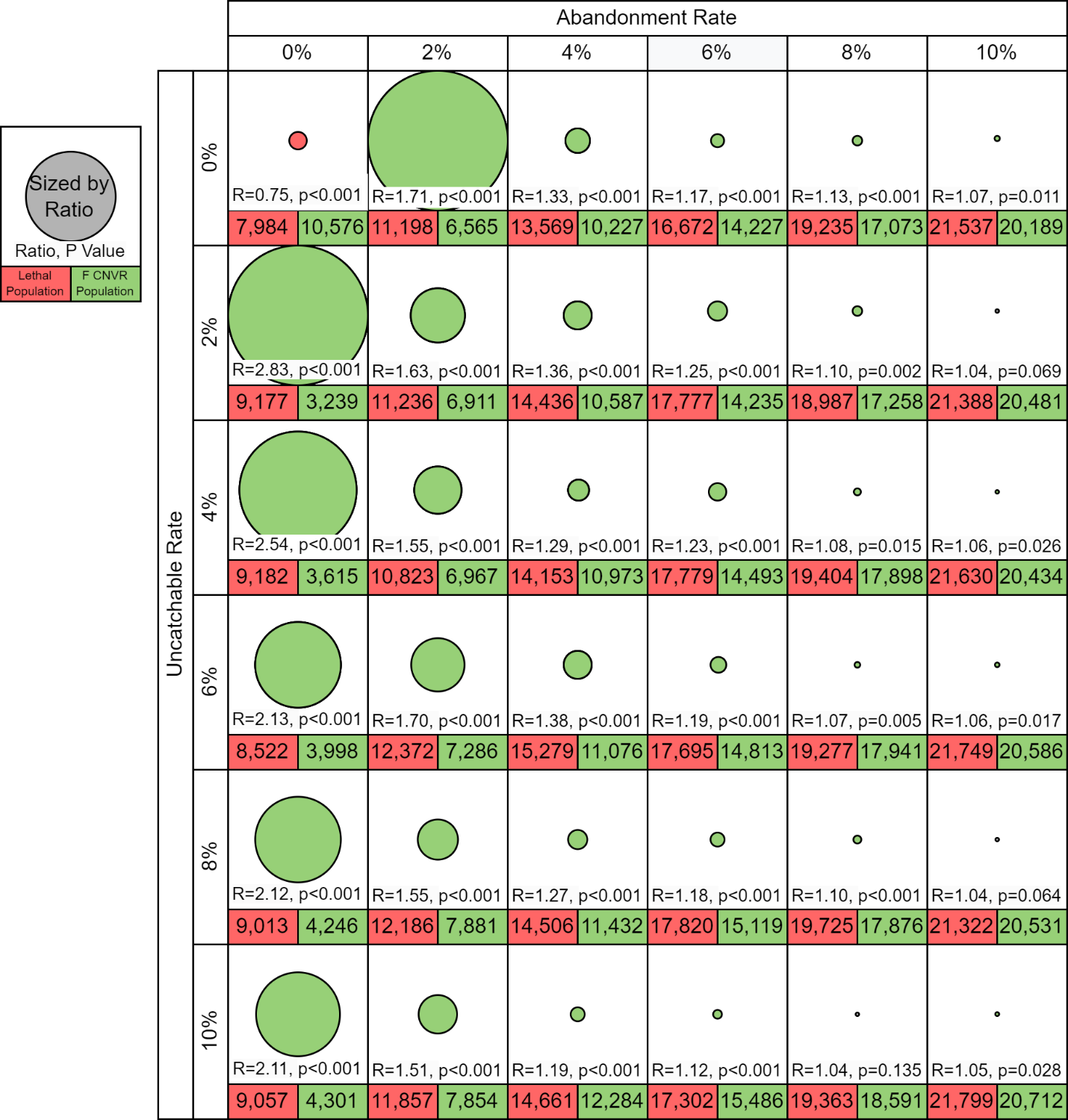


**Supplementary Figure 13**. A comparison of the mean dog population size at the end of 10 years of intervention (preceded by 5 years of non-intervention burn in) with variable abandonment rates and uncatchable percentages. The mean population size is listed for Lethal Control / Fertility control and below is their ratio and one-way ANOVA significance. Comparisons are colored by what management strategy produces the lower population and shaded by the ratio (grey when non-significant).


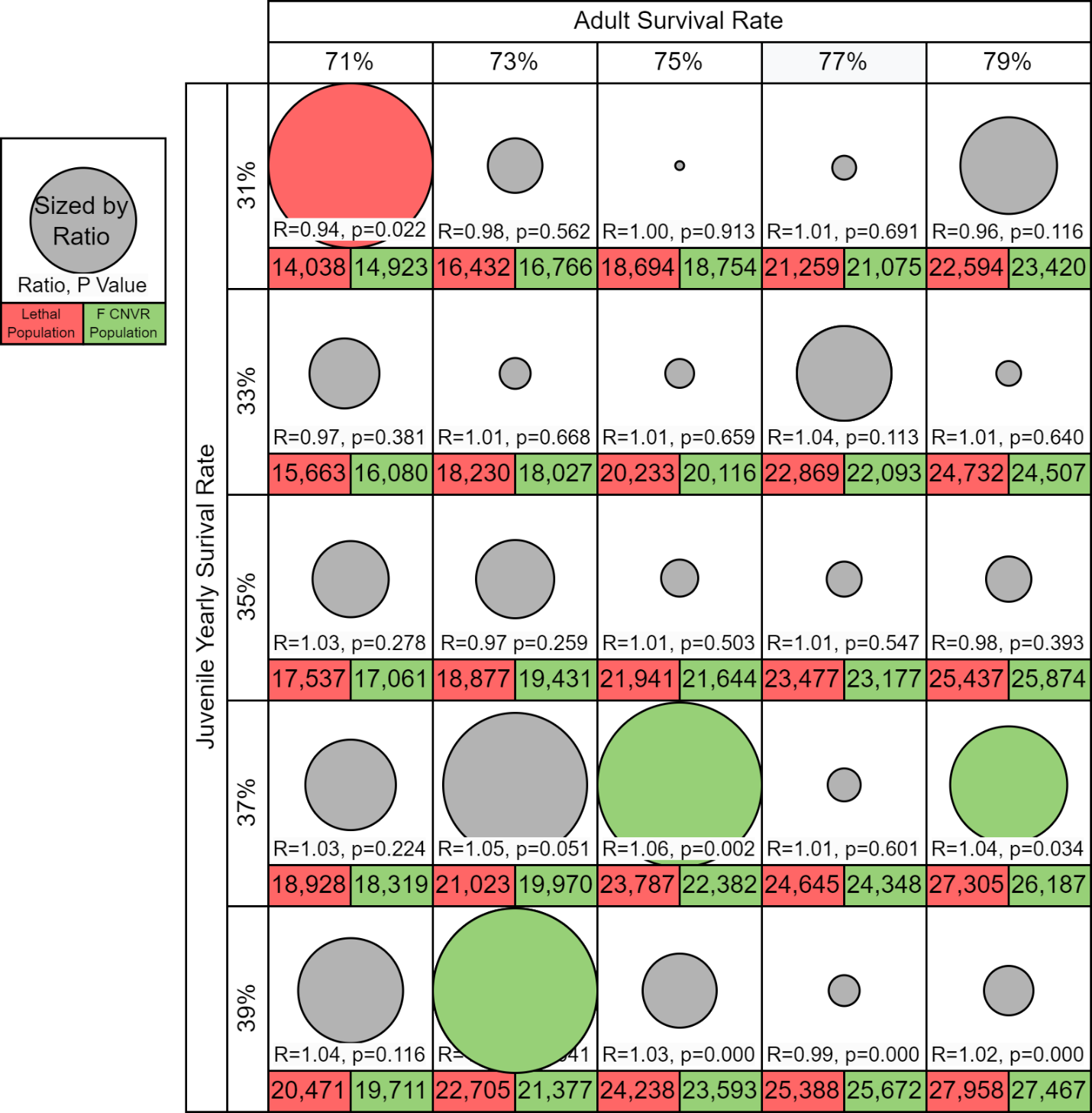


**Supplementary Figure 14.** A comparison of the mean dog population size at the end of 10 years of intervention (preceded by 5 years of non-intervention burn in) with variable adult and juvenile survival rates. The mean population size is listed for Lethal Control / Fertility control and below is their ratio and one-way ANOVA significance. Comparisons are colored by what management strategy produces the lower population and shaded by the ratio. Grey color indicates no statistically significant differences.


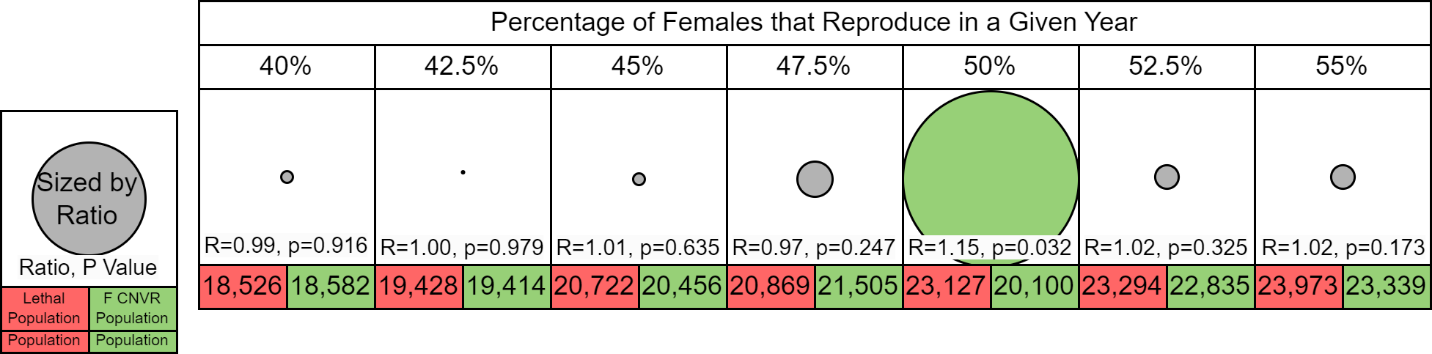


**Supplementary Figure 15.** A comparison of the mean dog population size at the end of 10 years of intervention (preceded by 5 years of non-intervention burn in) with modified reproductive rates. The mean population size is listed for Lethal Control / Fertility control and below is their ratio and one-way ANOVA significance. Comparisons are colored by what management strategy produces the lower population and shaded by the ratio. Grey color indicates no statistically significant differences.

The sensitivity analysis shows a clear trend in abandonment and uncatchability, as when both of these population drivers decline (fewer dogs are added by non-reproductive methods and fewer dogs are un-reachable to any intervention) the relative effectiveness of female-only CNVR and Lethal Control shifts the clear benefit to the fertility control method. Survival rates of adults and juveniles, in addition to the overall reproductive rate do not show a strong trend towards preferring any one management strategy.

**Additional Sensitivity Investigations**

Our ‘standard’ model structure assumes generally conservative estimates of the ability to vaccinate, including the following parameterizations:

- 10% of dogs are uncatchable.
- Dogs with an age of 6 months or older are caught for intervention (this is considered conservative because of the high mortality rate of dogs below 1 year of age, hence a potential to ‘waste’ a vaccine in terms of maximum vaccination coverage).

To test these assumptions, we constructed a ‘less conservative’ model parameterization with the following parameters:

- 2% of dogs are uncatchable.
- Dogs are only caught if they are over 12 months or older.

Both versions use the normal parameters of:

- Female-only CNVR scheme
- 35% Juvenile Survival
- 75% Adult Survival
- 10% Abandoned
- 30,000 Carrying Capacity
- 300 dogs caught per month

At the end of intervention, we compared the maximum vaccination coverage and the lapsed immunity percentage between these two model parameterizations using a one-way ANOVA (Supplementary Figure 16). We found that the standard model and less conservative model had no significant differences in the maximum vaccination coverage (mean = 27.10% vs 27.97%, p=0.372) or lapsed immunity percentage (mean = 12.91% vs 12.68%, p=0.464). These results show that the model is not significantly driven by these assumptions and should be robust to even larger changes in baseline parameters.


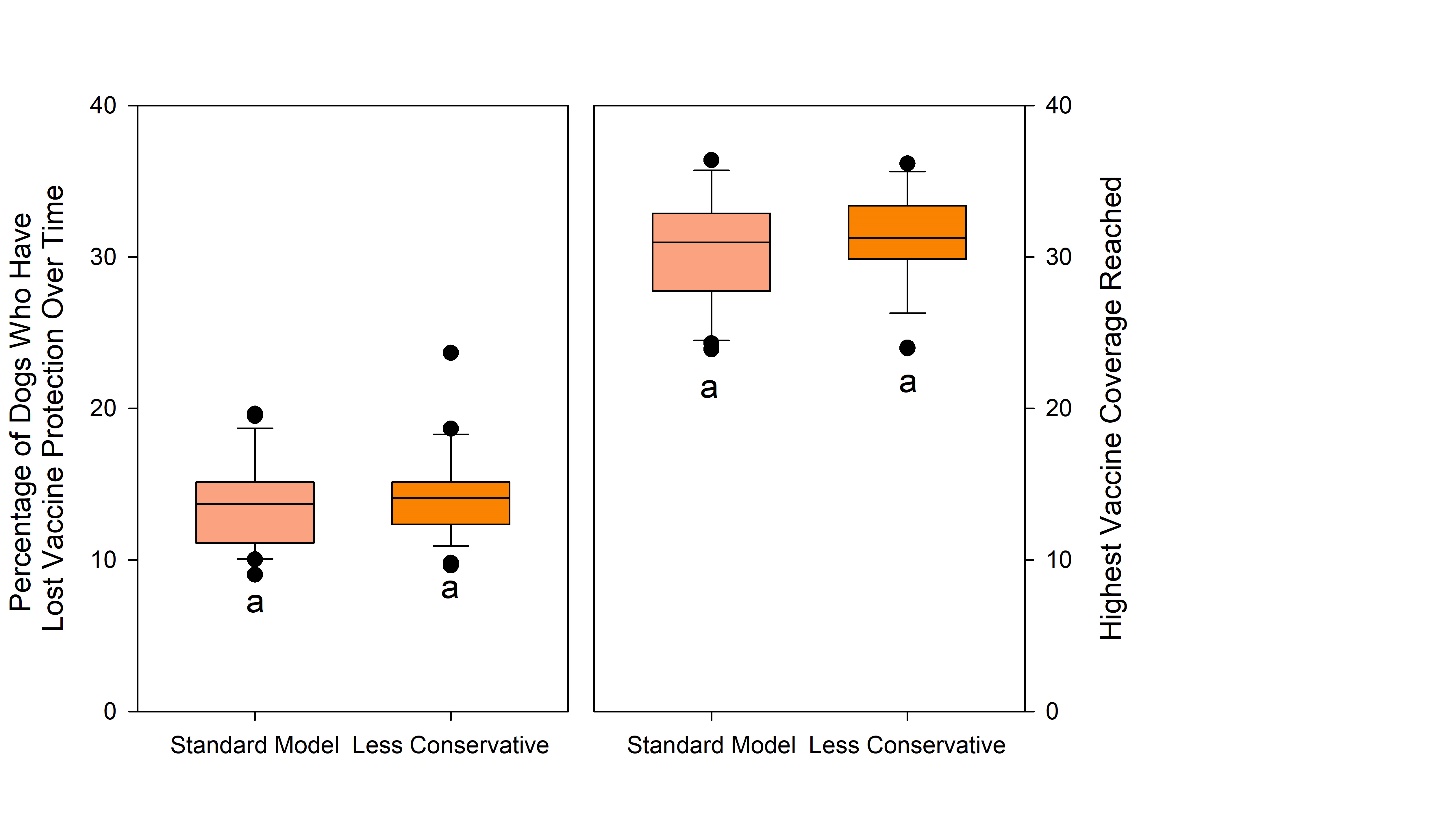


**Supplementary Figure 16.** The differences between the standard model and less conservative assumptions model on the maximum vaccination coverage and the lapsed immunity percentage.

We also compared the effect of capping effort in the intervention effort limiting submodel (Supplementary Figure 17).

Both versions use the standard parameters of:

- 35% Juvenile Survival
- 75% Adult Survival
- 10% Abandoned
- 30,000 Carrying Capacity

But differ in if the intervention effort limiting control is in place (stopping when a maximum driving distance is reached) or not (catching a minimum of 300 dogs per day, no matter how far you must travel to reach them).

We compared lagging mean dog population after 10 years of intervention using a one-way ANOVA. We found that the lethal control with no effort capping was significantly different than all other models with no other significant differences (a mean of 17,149.5 vs 20,751.1 average for the other three schemes, p<0.0001). This shows that lethal control benefits significantly more than CNVR from an artificial capture system where captures don’t decrease over time as difficulty increases.
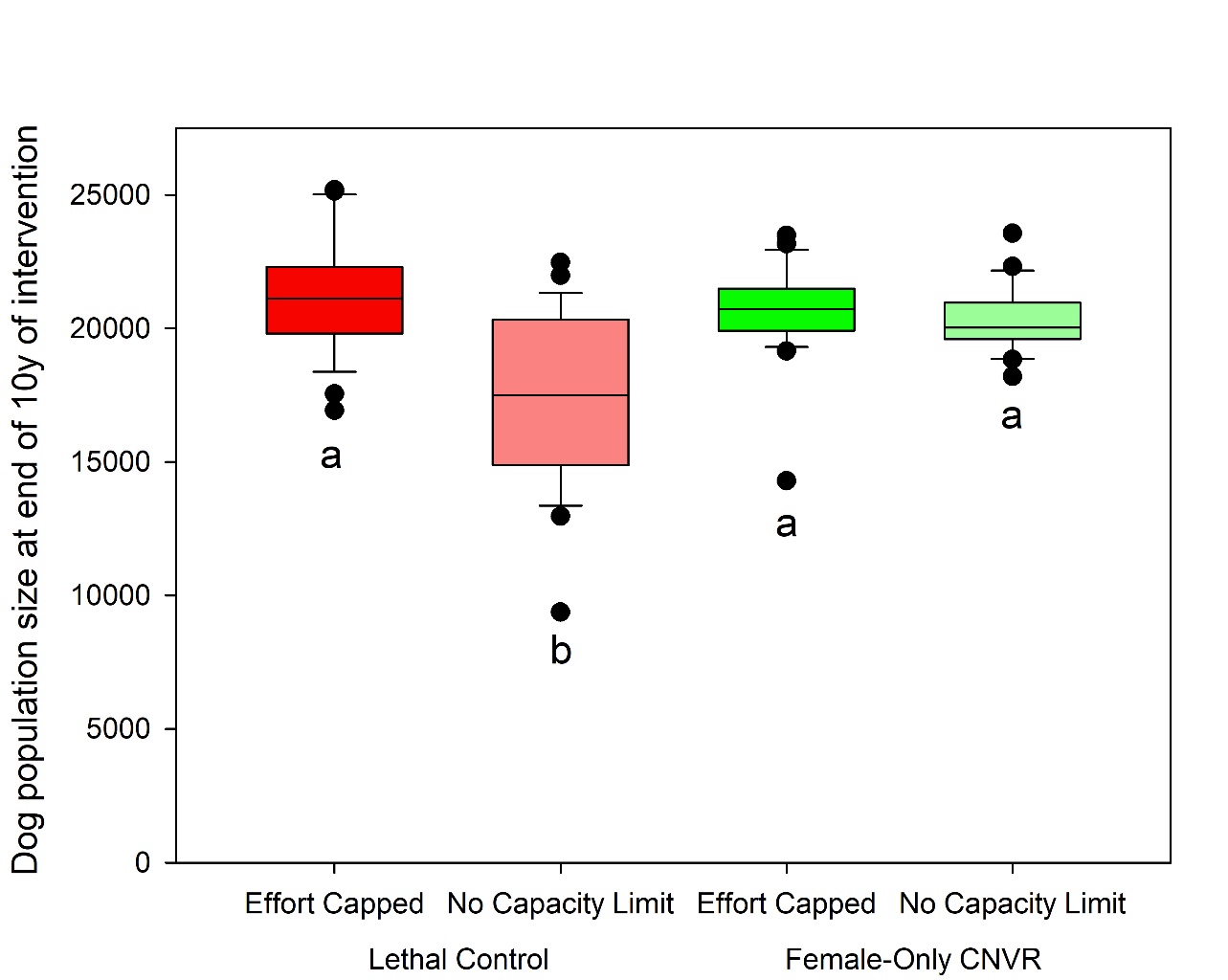


Supplementary Figure 17. The differences between the limiting the collection effort or not on both lethal and female-only CNVR on the final dog population after 10 years of intervention.

**Works Cited**

1. Wilensky, U. Netlogo. *http://ccl.northwestern.edu/netlogo/* (1999).

2. Grimm, V. *et al.* The ODD protocol for describing agent-based and other simulation models: A second update to improve clarity, replication, and structural realism. *Journal of Artificial Societies and Social Simulation* **23**, (2020).

3. Yoak, A. J., Reece, J. F., Gehrt, S. D. & Hamilton, I. M. Optimizing free-roaming dog control programs using agent-based models. *Ecological Modelling* **341**, 53–61 (2016).

4. Belsare, A. & Vanak, A. T. Modelling the challenges of managing free-ranging dog populations. *Scientific Reports* **10**, 1–12 (2020).

5. Reece, J. F., Chawla, S. K., Hiby, E. F. & Hiby, L. R. Fecundity and longevity of roaming dogs in Jaipur, India. *BMC Veterinary Research* **4**, 1–7 (2008).

6. Chawla, S. K. & Reece, J. F. Timing of oestrus and reproductive behaviour in Indian street dogs. *The Veterinary Record* **150**, 450 (2002).

7. Diaz, N. M. *et al.* Dog overpopulation and diagnosis of intestinal parasites on Santa Cruz Island, Galapagos 2016. *Preventive Veterinary Medicine* **157**, 99–104 (2018).

8. Shamsaddini, S. *et al.* Dynamic modeling of female neutering interventions for free-roaming dog population management in an urban setting of southeastern Iran. *Scientific Reports* **12**, 4781 (2022).

9. Nasiry, Z., Mazlan, M., Noordin, M. M. & Mohd Lila, M. A. Evaluation of Dynamics, Demography and Estimation of Free-Roaming Dog Population in Herat City, Afghanistan. *Animals* **13**, 1126 (2023).

10. Evans, M. *et al.* Free-roaming dog population dynamics in Ranchi, India. *Research in Veterinary Science* **143**, 115–123 (2022).

11. Reece, J. F. & Chawla, S. K. Control of rabies in Jaipur, India, by the sterilisation and vaccination of neighbourhood dogs. *Veterinary Record* **159**, 379–383 (2006).
